# Strategies for five tumour markers in the screening and diagnosis of female breast cancer

**DOI:** 10.3389/fonc.2022.1055855

**Published:** 2023-01-23

**Authors:** Jun Luo, Jianbo Xiao, Yuwei Yang, Gang Chen, Dong Hu, Jiawei Zeng

**Affiliations:** Department of Clinical Laboratory, Mianyang Central Hospital, School of Medicine, University of Electronic Science and Technology of China, Mianyang, China

**Keywords:** breast cancer, AFP, CEA, CA125, CA153, CA199

## Abstract

**Objective:**

This study evaluated the diagnostic value of different combinations of five commonly used tumour markers and screened the best combination of tumour markers.

**Methods:**

Regression analysis was used to evaluate 185 patients with suspected breast cancer admitted to Mianyang Central Hospital from January 2020 to December 2021. The differences of five tumour markers between a breast cancer group and a benign lesion group were analysed. The sensitivity and specificity of five tumour markers were compared.

**Results:**

Of 185 patients with suspected breast cancer, 108 patients had breast cancer and 77 patients had benign breast tumours. The detection results of carcinoembryonic antigen (CEA), alpha fetoprotein (AFP), carbohydrate antigen 125 (CA125), carbohydrate antigen 199 (CA199) and carbohydrate antigen 153 (CA153) in patients with breast cancer were significantly higher than those in patients with benign breast tumours. In the analysis of the single-detection results of tumour markers, CEA had the highest sensitivity (23.94%), CA153 had the highest specificity (96.43%), AFP had the highest accuracy (47.66%) and CA153 had the highest area under the curve (AUC) value (0.727). With the increase of parallel indicators, the sensitivity, accuracy and AUC value increased in turn, and the increase was obvious in the front. The increase began to slow down after the three parallel indicators. Among the different combinations of three parallel detections of breast cancer tumour markers, the highest sensitivity was AFP + CEA + CA153 (83.46%), the highest accuracy was AFP + CEA + CA153 and AFP + CA153 + CA125 (80.25%), and the highest AUC was CEA + CA125 + CA199 (0.922).

**Conclusion:**

AFP, CA153 and CA199 are recommended for clinical diagnosis of breast cancer. In routine physical examination and early breast cancer screening, the optimal combination of AFP + CEA + CA153 three parallel tests is recommended.

## 1 Introduction

The latest ‘2020 Global Cancer Report’ released by the World Health Organization/International Agency for Cancer Research shows that there are 2.2 million new cases of breast cancer in the world, more than the 2.2 million cases of lung cancer. Breast cancer has replaced lung cancer to become the world’s most frequent type of cancer. At the same time, 680,000 breast cancer deaths in 2020 were at the top of the list of global female cancer deaths (1). In 2020, 420,000 new cases of breast cancer were the first among the new cases of female cancer in China (1). In recent years, the incidence of breast cancer has gradually increased and showed a trend of occurrence in young people (2). In order to effectively reduce the mortality rate of patients and improve women’s quality of life, how to detect breast cancer early in patients and conduct early treatment has become a hot topic in clinical practice (3, 4). At present, the clinical screening and diagnosis methods of breast cancer mainly include imaging, ultrasound, pathology and detection of serum tumour markers. It was found that imaging, pathology and ultrasound examination were greatly affected by subjective factors, such as medical experience and technology. At the same time, conventional B ultrasound and key targets were less sensitive to small breast cancer lesions, which also affected the early detection and diagnosis of the disease.

It has been reported in relevant literature abroad that carcinoembryonic antigen (CEA) is a broad-spectrum tumour marker good for the evaluation of curative effect, condition and prognosis of breast cancer (5, 6). Studies have shown that common tumour markers, such as CEA, alpha fetoprotein (AFP), carbohydrate antigen 125 (CA125), carbohydrate antigen 199 (CA199) and carbohydrate antigen 153 (CA153), can make up for the deficiency in imaging to some extent (7, 8). The detection of serum tumour markers is characterised by convenient clinical development, easy acquisition of test specimens and low cost, and is one of the most widely carried out detection indexes in clinical laboratories. However, at present, there is either low sensitivity or low specificity in the application of any clinical marker alone, which cannot meet the demand of clinical application (9). The detection of single markers has limitations, and the optimal combination for detection of markers in each group has not yet been determined.

In this study, the sensitivity, specificity, positive predictive value, negative predictive value and diagnostic coincidence rate of five commonly used tumour markers in breast cancer patients were investigated. Screening involved the best combination of different conditions to provide detection strategies for early screening and auxiliary diagnosis of breast cancer using tumour markers.

## 2 Materials and methods

### 2.1 Research subjects

This study was a retrospective observational study. A total of 185 patients with suspected breast cancer admitted to Mianyang Central Hospital from January 2020 to December 2021 were selected as research objects by the convenient sampling method. Patients with breast cancer (group A) and patients with benign lesions (group B) were grouped according to pathological results.

Inclusion criteria of group A: (1) other malignant tumours and gynaecological diseases or benign breast lesions in patients, (2) breast cancer patients in line with the ‘Guidelines and norms for diagnosis and treatment of breast cancer’ (2021) (10).

Exclusion criteria of group A: (1) other malignant tumours and gynaecological diseases; (2) patients with severe diseases, such as those of the liver, kidney or heart; (3) pregnant or lactating women.

Inclusion criteria of group B: patients diagnosed with pathologically benign breast lesions (such as breast fibroadenoma).

Exclusion criteria of group B: (1) patients with malignant tumours or gynaecological diseases; (2) patients with severe diseases, such as those of the liver, kidney or heart; (3) pregnant or lactating women.

In this study, the patients and their families were informed of the risks and benefits during the process of participation. The relevant examination results were desensitised. The patients and their families signed their informed consent after fully understanding the relevant advantages and disadvantages. This study was approved by the ethics committee of the hospital (Approval number: 2022-MZ-0382-023).

### 2.2 Research methods

#### 2.2.1 Detection method

First, 3 mL of venous blood was taken from the two groups of patients on an empty stomach in the morning. After standing and self-coagulation, the Boko TGL-30M (30,000 r/rain) desktop ultra-speed freezing centrifuge (China, Shangdong, Boko Medical Devices Co., Ltd.) was used for continuous centrifugation for 10 min. The serum was separated and stored in a refrigerator at –20°C for testing. Second, the concentrations of AFP, CEA, CA153, CA125 and CA199 were detected by Siemens automatic chemiluminescence immunoassay analyser ADVIA Centaur XP (China, Siemens Medical Diagnostic Products [Shanghai] Co., Ltd.). Matching test reagents were used, and in strict accordance with the kit instructions for testing operations, with indoor quality control.

The sensitivity, specificity, positive predictive value and negative predictive value of AFP, CEA, CA153, CA125 and CA199 tumour markers in group A and group B were analysed with pathological examination as the gold standard. The differences in sensitivity, specificity, positive predictive value, negative predictive value and diagnostic accordance rate were analysed by different combinations of the five tumour markers (2, 3, 4 and 5).

#### 2.2.2 Diagnostic criteria

The reference range of each index is as follows: AFP ≤ 7.29 ng/mL, CEA < 5.00 ng/mL, CA153 < 31.30 U/mL, CA125 < 35.00 U/mL, CA199 < 37.00 U/mL. Each index detection value is higher than the reference interval is positive. When each index is detected jointly, any index is judged positive when it exceeds the reference range.

#### 2.2.3 Statistical analysis method

All parameters were analysed using IBM SPSS 23.0 statistical analysis software. Using Shapiro–Wilk test measurement data, numerical variables are in line with normal distribution, as described by ‘mean ± standard deviation’ (X ± S). Independent sample t test was used for comparative analysis between the two independent samples. At the same time, the sensitivity, specificity, accuracy and area under the curve (AUC) values of single detection and combined detection of the five tumour markers were calculated. The receiver operating characteristic curve and related statistical charts were drawn by GraphPad Prism 5.0 software and SPSS 23.0 software. *P* < 0.05 indicated that the difference was statistically significant.

## 3 Results

### 3.1 Basic information and clinical features

A total of 185 subjects were enrolled, including 108 patients with breast cancer (group A) and 77 patients with benign breast tumours (group B). The age of group A participants was 33–67 years old, with an average of 42.25 ± 7.37 years old. The age of group B participants was 32–65 years old, with an average of 43.33 ± 7.52 years old. The body weight of group A participants was 50.57 ± 7.82 kg, and their body mass index was 22.58 ± 3.98. The weight of group B was 51.37 ± 8.29 kg, and their body mass index was 21.08 ± 3.77. There was no significant difference in age, weight or body mass index between the two groups, suggesting that the two groups were comparable. There were 108 cases of breast cancer patients as classified by the international tumour-node-metastasis classification of breast cancer published by the international anti-cancer alliance for clinical staging. There were 64 cases of patients with breast cancer I and II, and 44 cases of breast cancer III and IV. Among 77 patients with benign breast tumours, 31 cases were fibroadenoma, 23 cases were lobular hyperplasia, 20 cases were breast cyst and three cases were of breast inflammation, as shown in **Table 1**.

**Table 1:** The basic information of the two patients.

Variable	Group A (n=108)	Group B (n=77)	*t*	*P*
Age(year)	42.25 ± 7.37	43.33 ± 7.52	1.238	0.193
Weight(Kg)	50.57 ± 7.82 kg	51.37 ± 8.29	1.314	0.082
BMI(Kg/m2)	22.58 ± 3.98	21.08 ± 3.77	0.924	0.347
TNM staging				
I/II	64	–		
III/IV	44	–		
Benign tumor type				
Fibroadenoma	–	31		
Lobular hyperplasia	–	23		
Breast cyst	–	20		
Inflammation	–	3		

BMI, Body mass index; TNM, The international tumor-node-metastasis.

### 3.2 Comparison of tumour markers between the two groups

The detection results of serum tumour markers AFP, CEA, CA153, CA125 and CA199 in group A were significantly higher than those in group B (*P* < 0.05). See **Table 2** for details.

**Table 2:** Comparison of tumor markers between group A and group B.

Tumor marker	Group A (n=108)	Group B (n=77)	*t*	*P*
AFP(ng/ml)	16.23 ± 3.04	4.01 ± 2.92	17.433	<0.0001*
CEA(ng/ml)	9.83 ± 3.44	5.15 ± 3.22	6.981	<0.001*
CA153(U/ml)	23.56 ± 11.63	10.46 ± 6.48	12.894	<0.001*
CA125(U/ml)	17.57 ± 7.87	12.53 ± 7.28	8.644	<0.001*
CA199(U/ml)	18.25 ± 8.67	11.63 ± 6.47	9.212	<0.001*

*The difference was statistically significant.

AFP, alpha fetoprotein; CEA, carcinoembryonic antigen; CA153, carbohydrate antigen 153; CA125, carbohydrate antigen 125; CA199, carbohydrate antigen 199.

### 3.3 Analysis of single-detection results of tumour markers

The sensitivity, specificity, accuracy, AUC and 95% confidence interval of tumour markers AFP, CEA, CA153, CA125 and CA199 in the diagnosis of breast cancer are shown in **Table 3**. The highest sensitivity was CEA (23.94%), and the lowest was CA153 (15.00%). The highest specificity was CA153 (96.43%), and the lowest was CEA (67.44%). The highest accuracy was AFP (47.66%), and the lowest was CEA (39.29%). The highest AUC was CA153 (0.727), and the lowest was CA199 (0.598).

**Table 3:** Results of single detection of 5 tumor markers.

Tumor marker	Sensitivity (%)	Specificity(%)	Accuracy (%)	AUC value	95%CI
AFP	22.22	94.33	47.66	0.627	0577~0.678
CEA	23.94	67.44	39.29	0.679	0.634~0.723
CA153	15.00	96.43	43.50	0.727	0.677~0.778
CA125	16.54	93.57	43.50	0.695	0.642~0.747
CA199	19.36	94.54	45.89	0.598	0.547~0.649

AUC, Area under the curve; AFP, alpha fetoprotein; CEA, carcinoembryonic antigen; CA153, carbohydrate antigen 153; CA125, carbohydrate antigen 125; CA199, carbohydrate antigen 199.

### 3.4 Analysis of detection results of tumour markers in different parallel modes

From the combination of one, two, three, four and five tumour markers, each group was tested in parallel.

**Figure 1:**
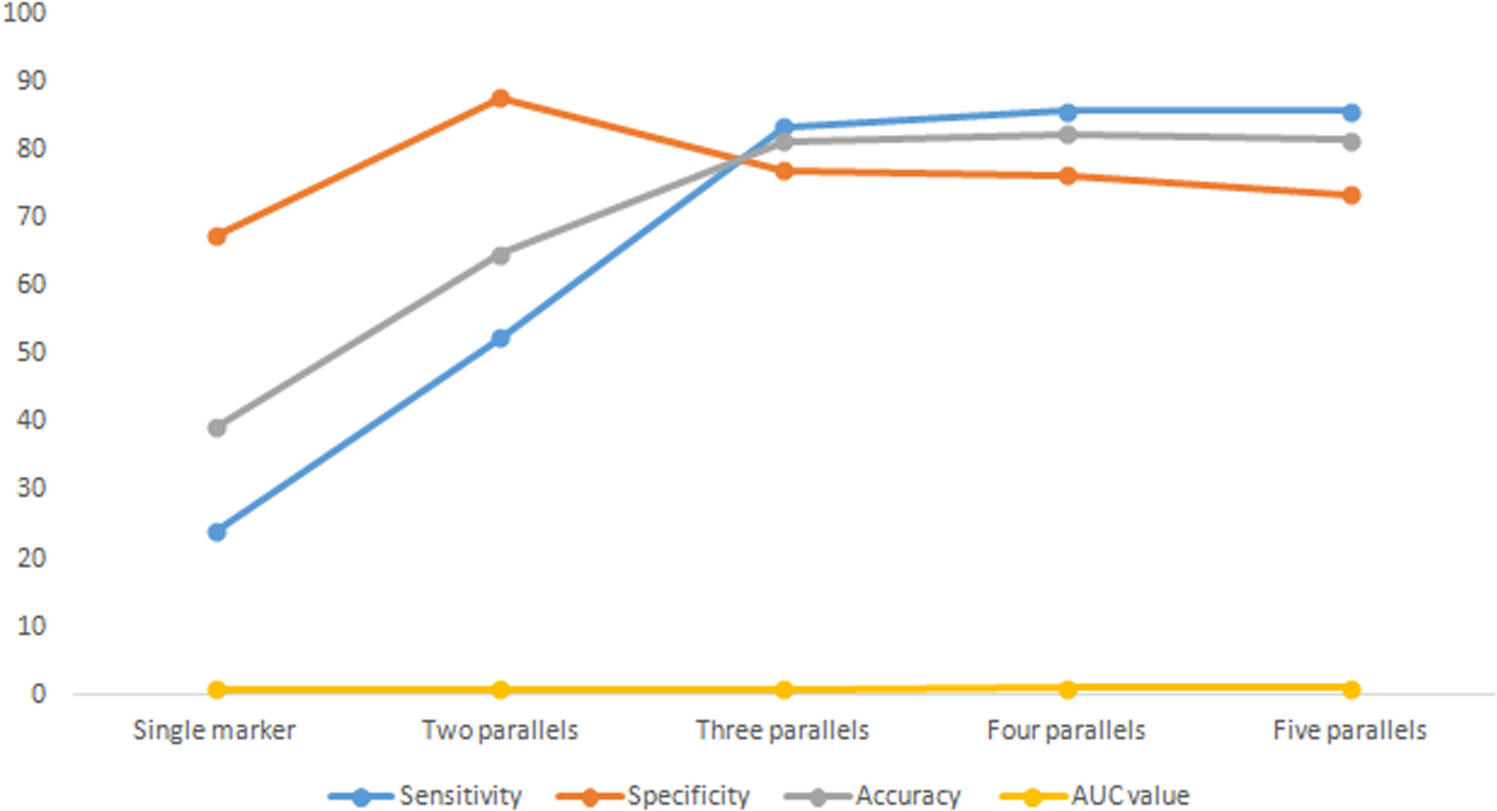
Diagnosis results of five tumor markers by different parallel methods.

The combination with the highest sensitivity was selected in the test, and the change trend of diagnostic indexes, such as sensitivity and specificity, was analysed. See **Table 4** and **Figure 1**. The results showed that with the increase of parallel indicators, the sensitivity, accuracy and AUC values increased in turn, and the increase was obvious in the front. The increase began to slow down after three parallels. Three parallels should be the optimal combination for the detection of breast cancer tumour markers.

**Table 4:** Diagnosis results of five tumor markers in different parallel ways (%).

Tumor markers	Sensitivity (%)	Specificity (%)	Accuracy (%)	AUC value	95%CI
Single item	23.94	67.44	39.29	0.679	0.634~0.723
two parallel	52.31	87.86	64.75	0.779	0.734~0.823
Three parallels	83.46	74.29	80.25	0.913	0.885~0.942
Four parallels	85.77	76.43	82.50	0.923	0.896~0.951
Five parallels	85.77	73.57	81.50	0.928	0.902~0.954

AUC, Area under the curve.

### 3.5 Analysis of detection results of three parallel methods for tumour markers

Among the different combinations of three parallel detections of breast cancer tumour markers, the highest sensitivity was AFP + CEA + CA153 (83.46%), the highest accuracy was AFP + CEA + CA153 and AFP + CA153 + CA125 (80.25%), and the highest AUC was CEA + CA125 + CA199 (0.922). Following comprehensive consideration, the optimal combination was AFP + CEA + CA153 three parallel detection (See **Table 5**).

**Table 5:** Analysis of Detection Results of Three Parallel Methods for Tumor Markers.

Tumor markers	Sensitivity (%)	Specificity(%)	Accuracy(%)	AUC value	95% CI
AFP+CEA+CA153	83.46	74.29	80.25	0.913	0.885~0.942
AFP+CEA+CA125	78.85	78.57	78.75	0.873	0.843~0.901
AFP+CEA+CA199	80.38	75.00	78.50	0.889	0.857~0.911
AFP+CA153+CA125	82.31	77.14	80.25	0.905	0.875~0.935
AFP+CA153+CA199	71.29	86.17	79.33	0.813	0.758~0.843
AFP+CA125+CA199	74.78	82.66	75.34	0.782	0.712~0.801
CEA+CA153+CA125	75.34	85.33	72.67	0.713	0.681~0.877
CEA+CA153+CA199	81.36	69.38	73.38	0.755	0.723~0.798
CEA+CA125+CA199	83.08	73.57	79.75	0.922	0.875~0.941
CA153+CA125+CA199	68.64	87.38	77.23	0.799	0.742~0.887

AUC, Area under the curve; AFP, alpha fetoprotein; CEA, carcinoembryonic antigen; CA153, carbohydrate antigen 153; CA125, carbohydrate antigen 125; CA199, carbohydrate antigen 199.

## 4 Discussion

### 4.1 Difference of five tumour markers between breast cancer group and benign lesion group

The results showed that there were significant differences in the levels of AFP, CEA, CA153, CA125 and CA199 between the breast cancer group and the benign lesion group. The levels of the five tumour markers in the breast cancer group were higher than those in the benign lesion group. The increases in AFP, CEA, CA153, CA125 and CA199 tumour markers is closely related to the occurrence of breast cancer, suggesting that five tumour markers are feasible for screening or auxiliary diagnosis of breast cancer.

AFP is a specific tumour marker for the diagnosis of primary liver cancer (11) but can show a high concentration when a variety of tumours occur. AFP detection is also valuable in the diagnosis of early breast cancer (12). When malignant tumours occur, the release of ferritin increases due to tumour infiltration and necrosis, while the ability of the liver to clear it decreases. The synthesis of tumour cells increases, resulting in an increase in its concentration.

CEA is a broad-spectrum tumour marker, which exists on the surface of cancer cells differentiated from endoderm cells. It is elevated in the serum of gastrointestinal tract, breast cancer, lung cancer and other malignant tumours. Serum CEA can be used as a biomarker for the diagnosis of colorectal cancer in clinic (13). Studies have reported that, although CEA has poor specificity in the diagnosis of other malignant tumours, it has important clinical value in the differential diagnosis of malignant tumours, disease monitoring and efficacy evaluation (14).

As a broad-spectrum tumour marker, CA125 is a glycoprotein on the cell surface with a high molecular weight (15). CA125 has been widely used in the diagnosis of ovarian cancer, fallopian tube cancer, endometrial cancer, cervical cancer, pancreatic cancer, liver cancer, lung cancer and digestive tumours in recent years. The single-detection sensitivity of CA125 in each piece of literature was different: 3.75%, 6.2%, 18%, 29% and 69.2%, respectively (16).

CA153 is a common monitoring factor in the diagnosis of breast cancer. It is often used as a non-specific marker of breast cancer and has important clinical significance in the differential diagnosis and monitoring of the curative effect of other malignant tumours. CA153 is a marker with high specificity for breast cancer, and its level in patients with breast cancer has increased to a certain extent (17).

CA199 is the most effective biomarker and an indicator of abnormal glycosylation of pancreatic cancer. CA199 plays a role as a biomarker, predictor and promoter in pancreatic cancer. As a biomarker, its sensitivity is about 80% (18). CA199 can be used for the diagnosis of metastatic breast cancer (6).

### 4.2 The impact of single-detection sensitivity, specificity and other indicators

In this study, the sensitivity of single tumour marker detection was low (15.00%–23.94%). All specificities, except that of CEA (67.44%), were above 90%, and their accuracy was less than 50%. The study included 185 subjects with a moderate sample size and was strictly controlled according to the inclusion and exclusion criteria; the five tumour markers were detected by Abbott automatic chemiluminescence immunoassay analyser. This new instrument, purchased in 2017, was tested by professional inspectors after training in accordance with the operating procedures. The results should be reliable. Five tumour marker single-test results suggested that only one or two tests, such as routine physical examination or early breast cancer screening, had little clinical significance. However, most specificities were very high, especially that of the tumour marker CA153. It had a specificity as high as 96.43%. Based on only this detection index, patients could be diagnosed with breast cancer. CA153, as an auxiliary diagnosis, has great clinical reference value.

### 4.3 Changes in diagnostic value of parallel test sensitivity, specificity and other indicators

At present, tumour markers are widely used, but the sensitivity and accuracy of single detection are not ideal. In view of this, this study intends to gradually increase the number of parallel tests of tumour markers to improve sensitivity, negative predictive value and diagnostic coincidence rate. The combined experiment found that with the increase in combined detection of tumour markers, the sensitivity increased rapidly to a flat phase, with the highest sensitivity being 85.77%. This can be used as a routine physical examination or a breast cancer screening method and can also be carried out with B ultrasound or platinum target in clinic. The specificity decreased gradually with an increase in the number of combined experiments. From the perspective of specificity and positive predictive value, the combined experiment was not meaningful. Clinicians can only refer to the results of single-index detection. The diagnostic accuracy improved. Detection fees also began to rise; each increased by 40 RMB. The three–three parallel test was the turning point. Based on the changes of the above indicators, when conducting a routine physical examination or breast cancer screening, considering the cost performance, it is recommended to use the top three of the three combinations: AFP + CEA + CA153 and AFP + CA153 + CA125, with had the highest accuracy (80.25%), or CEA + CA125 + CA199, which had the highest AUC value (0.922). Following comprehensive consideration, the optimal combination is AFP + CEA + CA153 three parallel detection.

In the past, there were similar studies to analyse the diagnostic value of tumour markers, but most of them were limited to the analysis of about four tumour markers. There were not five common tumour markers, especially no evaluation of the combined experimental effect of five tumour markers. However, this study is retrospective and prone to selection bias. Due to lack of time, it is not possible to classify the course of breast cancer in the study subjects. In the future, we will continue to explore the changes of tumour markers in different courses of breast cancer, evaluate the efficacy of breast cancer surgery, chemotherapy, radiotherapy or endocrine therapy and predict the prognosis of breast cancer recurrence or metastasis and five-year survival rate.

## 5 Conclusion

AFP, CA153 and CA199 are recommended for clinical diagnosis of breast cancer. In routine physical examination and early breast cancer screening, the optimal combination of AFP + CEA + CA153 three parallel tests is recommended.

## Data availability statement

The original contributions presented in the study are included in the article/supplementary material. Further inquiries can be directed to the corresponding author.

## Ethics statement

The studies involving human participants were reviewed and approved by Ethics committee of Mianyang Central Hospital. The patients/participants provided their written informed consent to participate in this study.

## Author contributions

JL and JWZ conceived of the study, and GC and DH participated in its design and data analysis and statistics and JBX and YWY helped to draft the manuscript. All authors contributed to the article and approved the submitted version.
